# Pulmonary exacerbations in insured patients with bronchiectasis over 2 years

**DOI:** 10.1183/23120541.00021-2023

**Published:** 2023-07-03

**Authors:** Patrick A. Flume, Joseph Feliciano, Matthew Lucci, Jasmanda Wu, Sebastian Fucile, Mariam Hassan, Anjan Chatterjee

**Affiliations:** 1 Medical University of South Carolina, Charleston, SC, USA; 2 Insmed Incorporated, Bridgewater, NJ, USA; 3 Panalgo, Boston, MA, USA

## Abstract

**Background:**

Patients with bronchiectasis experience persistent symptoms and frequent pulmonary exacerbations; this study investigated the frequency of exacerbations and all-cause hospitalisation.

**Methods:**

This longitudinal, retrospective, claims database study (IBM® MarketScan®) identified patients aged ≥18 years from 1 July 2015 through 30 September 2018. Exacerbations were identified by bronchiectasis inpatient claim or a healthcare interaction, followed by antibiotic prescription within 7 days. Patients with ≥36 months of continuous health plan enrolment (12 months preceding the first bronchiectasis claim, *i.e.*, baseline period and ≥24 months of follow-up) were included. Patients with cystic fibrosis at baseline were excluded. A multivariable logistic regression model identified baseline factors associated with having ≥2 exacerbations over the 2-year follow-up period.

**Results:**

In total, 14 798 patients with bronchiectasis were identified; 64.5% were female, 82.7% were aged ≥55 years and 42.7% had ≥2 exacerbations at baseline. Having ≥2 exacerbations after 2 years was positively associated with chronic macrolide use, long-acting β2 agonist use, gastro-oesophageal reflux disease, heart failure and *Pseudomonas aeruginosa*. Frequent exacerbations (≥2) at baseline were significantly associated with greater likelihood of experiencing ≥2 exacerbations during the first and second year's follow-up (unadjusted odds ratios 3.35 (95% CI 3.1–3.6) and 2.96 (95% CI 2.8–3.2), respectively). The proportion of patients experiencing ≥1 all-cause hospitalisation cumulatively increased from 41.0% in the first year of follow-up to 51.1% over 2 years' follow-up.

**Conclusion:**

Frequent exacerbations in patients with bronchiectasis may increase the likelihood of future exacerbations over 2 years of follow-up, with increased hospitalisation rates over time.

## Abstract 2

**In this longitudinal, retrospective, claims database study, frequent exacerbations in patients with bronchiectasis increased the likelihood of subsequent exacerbations and hospitalisations, highlighting the importance of preventing exacerbation** https://bit.ly/44207At

## Introduction

The chronic respiratory condition bronchiectasis is a heterogeneous disease characterised by permanent dilation of the bronchi, persistent cough and recurrent respiratory infections [1–3]. Estimates of the prevalence of bronchiectasis vary but are reportedly increasing globally [4–6]. In 2013, prevalence in the USA was estimated to be 213 cases per 100 000 persons [5]. Most patients with bronchiectasis endure daily symptoms of cough and sputum production, and some experience episodes of worsening of these symptoms, typically called pulmonary exacerbations [1].

Pulmonary exacerbations result in greater airway and systemic inflammation and are associated with the progression of bronchiectasis, worse quality of life and other comorbidities such as gastro-oesophageal reflux disease (GERD) [1]. Ultimately, higher numbers of exacerbations are associated with accelerated decline in lung function [7] and increased risk of mortality [1, 3, 8]. The European Respiratory Society's guidelines for bronchiectasis establish exacerbation prevention as one of the objectives of bronchiectasis treatment and recommend treatment with long-term macrolides and inhaled antibiotics for adults with frequent (*e.g.*, two to three or more) exacerbations each year [3]. However, patients continue to experience frequent pulmonary exacerbations, despite antibiotic treatment.

Pulmonary exacerbations are associated with a greater number of emergency department (ED) visits and increased rates of hospitalisation, leading to increased hospitalisation costs and considerable economic burden [1, 3, 9–12]. A systematic literature review indicated that bronchiectasis has a significant economic burden driven by hospitalisation costs, particularly for patients with frequent pulmonary exacerbations [11]. However, these studies may have underestimated the frequency of pulmonary exacerbations and disease progression, as exacerbations were identified using *International Classification of Diseases, Ninth Revision* (ICD-9) codes only and evaluated pulmonary exacerbations over a 1-year follow-up period [9–11].

Therapeutic interventions that can prevent patients from experiencing exacerbations over time are of potential clinical importance for improvement in quality of life and the preservation of lung function, in addition to reducing the associated economic burden. An understanding of the factors associated with pulmonary exacerbations is also needed to identify patients who may be best suited to preventative therapies.

This longitudinal, retrospective, claims database study used an algorithm to define exacerbations based on inpatient or outpatient claims and antibiotics prescription. We evaluated pulmonary exacerbation frequency and all-cause hospitalisation frequency in patients with bronchiectasis over 1 and 2 years of follow-up, as well as baseline factors associated with having ≥2 exacerbations.

## Methods

### Data collection

Data were derived from the IBM**®** MarketScan**®** Commercial Claims and Encounters and Medicare Supplemental Core databases (hereinafter, the “MarketScan database”), a large healthcare claims repository that captures medical and drug data from employers and health plans for over 24.5 million individuals [13]. Data from patients with Medicare are captured while they are still employed and using a commercial plan [13]. The Medicare data encompass employees, spouses and dependants who are covered by employer-sponsored private health insurance in the USA [13].

### Study population

**FIGURE 1:**
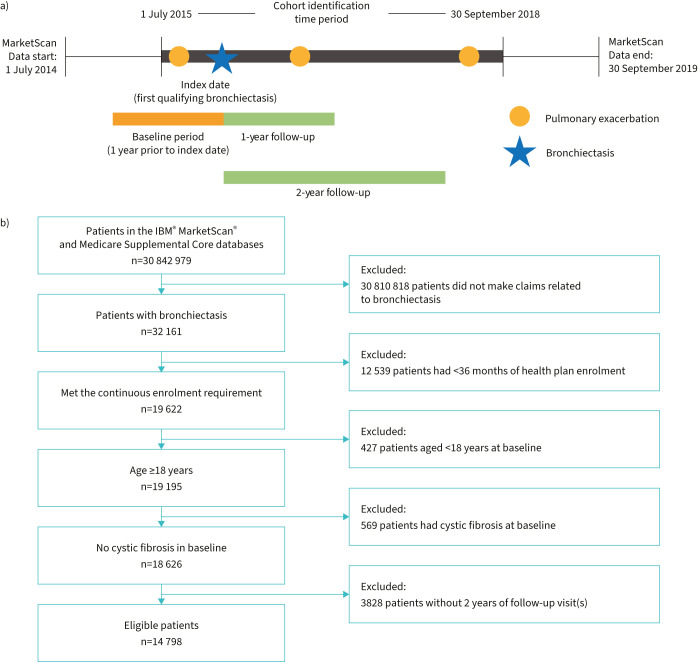
a) Study design diagram showing the key events from the start to the end of study. Patients with bronchiectasis were selected from the MarketScan database during the cohort identification period. The first qualifying bronchiectasis claim was the index date; the year prior to index was the baseline period and the 2 years after index were the follow-up period. b) STROBE (Strengthening the Reporting of Observational Studies in Epidemiology) flow diagram showing eligibility for analysis.

Patients with bronchiectasis ≥18 years of age were identified in the MarketScan database from 1 July 2015 through 30 September 2018 (figure 1). Patients must have had ≥36 months of continuous health plan enrolment, including 12 months preceding the first bronchiectasis claim (*i.e.*, baseline period) and ≥24 months of follow-up. Patients met the criteria of having bronchiectasis if they had one or more of the following: ≥2 outpatient claims for bronchiectasis occurring ≥30 days apart, high-resolution computed tomography scan followed by two subsequent claims for bronchiectasis (one claim occurring ≤60 days after the computed tomography scan) or ≥1 hospitalisation with a bronchiectasis diagnosis. Patients with a diagnosis of cystic fibrosis during the 1-year baseline were excluded.

### Data analysis

Patient characteristics, comorbidities of interest and healthcare resource use were summarised descriptively. Demographics were measured on the index date starting from 1 July 2015. Other baseline characteristics were measured during the 1-year baseline period. Comorbidities were required to be on ≥2 separate outpatient claims, >30 days apart, or a diagnosis on one inpatient hospitalisation claim during the 1-year baseline period. Chronic macrolide/antibiotic use was defined as having at least 90 total treated days. Repeat antibiotic use was defined as using the same antibiotic for 2 consecutive months. Patients met the criteria for having a pulmonary exacerbation if they had an inpatient claim with a bronchiectasis diagnosis, or a healthcare office visit with a bronchiectasis diagnosis followed by a prescription for macrolide, amoxicillin/clavulanic acid or fluoroquinolone antibiotics within 7 days. Pulmonary exacerbation events were not included as bronchiectasis-related events if the primary claim was otitis media, acute rhinosinusitis or COPD. The number of pulmonary exacerbations and all-cause hospitalisation frequency are reported categorically as a proportion of patients experiencing 0, 1, 2 or ≥3 events over the first and second year of follow-up. First year of follow-up was the period between the index date and 12 months after. The second year was the period from the end of the first year through 12 months after. All-cause ED visits are reported as mean and median occurrences during the 1-year baseline period. For the purpose of this analysis, frequent exacerbations were defined as patients experiencing ≥2 exacerbations per year, to establish a threshold for patients who experience multiple exacerbations compared with those who do not.

### Statistical analyses

To determine the p-value of continuous variables, t*-*tests were used. The Pearson chi-squared test was used for binary and categorical variables, apart from lung transplant comorbidity, which used the Fisher exact test due to the small, expected sample size.

Unadjusted odds ratios (ORs) with 95% confidence intervals (CIs) were derived for the cross-tabulation of number of exacerbations (≥2; yes *versus* no) during the study observation periods (baseline, follow-up year 1 and follow-up year 2). The OR was derived from the ratio of the probability of having ≥2 exacerbations during the baseline period or year 1 of follow-up and the probability of exacerbations in the second year of follow-up:

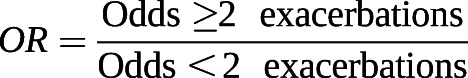

A multivariable logistic regression model was fit to assess if selected baseline factors were significantly associated with having ≥2 exacerbations in the 2-year period following the bronchiectasis index date. Covariates were selected through a parsimonious analysis assessing statistical differences in baseline demographics and clinical characteristics, comparing those who had ≥2 exacerbations *versus* those who had <2 exacerbations in the 2-year follow-up period. Input was then collected from expert clinicians to provide recommendations on defining covariates. For example, medications used to manage respiratory conditions were used as a proxy for respiratory comorbidities such as asthma and COPD, due to the availability of this information in the databases. Using drug claims as proxy minimised the impact of errant ICD-9/10 coding given these data are firstly for administrative purposes. Variance inflation factors (VIFs) were calculated among the final set of model covariates to assess multicollinearity. The specific covariates used in the final model included the following variables at baseline: age at index, sex, number of ED visits, exacerbations, nontuberculous mycobacterial (NTM) disease, chronic macrolide use, long-acting β2 agonist (LABA) use, inhaled steroid use, cough, dyspnoea, GERD, heart failure, haemoptysis and the presence of *Pseudomonas aeruginosa*. Outcome modifiers for the multivariable analysis were also identified as an effort to avoid bias.

## Results

### Study population and patient characteristics at baseline

A total of 14 798 patients with bronchiectasis and 2 years of follow-up were identified (figure 1). Most patients were female (64.5%), ≥55 years of age (82.8%) and eligible for Medicare supplemental insurance (54.0%) (table 1). Demographic characteristics were mostly well balanced, and no significant differences were noted in age or payer when patients were stratified by number of exacerbations over the entire 2-year follow-up period (table 1).

**TABLE 1:** Baseline demographics at index date stratified by exacerbations over the 2-year follow-up period

	**<2 exacerbations**	**≥2 exacerbations**	**Overall**	**p-value**
**Patients n**	6029	8769	14 798	
**Age years**				0.1587
Mean±sd	66.8±14.4	66.5±14.3	66.6±14.4	
Median (IQR)	66 (58–78)	67 (58–77)	67 (58–78)	
**Age group years**				0.0527
18–34	139 (2.3)	235 (2.7)	374 (2.5)	
35–54	906 (15.0)	1276 (14.6)	2182 (14.8)	
55–69	2332 (38.7)	3421 (39.0)	5753 (38.9)	
70–84	1982 (32.9)	2974 (33.9)	4956 (33.5)	
≥85	670 (11.1)	863 (9.8)	1533 (10.4)	
**Female sex**	3785 (62.8)	5758 (65.7)	9543 (64.5)	0.0003
**Region**				0.0064
Midwest	1280 (21.2)	1771 (20.2)	3051 (20.6)	
Northeast	1633 (27.1)	2233 (25.5)	3866 (26.1)	
South	2162 (35.9)	3396 (38.7)	5558 (37.6)	
West	724 (12.0)	1015 (11.6)	1739 (11.8)	
Missing	230 (3.8)	354 (4.0)	584 (4.0)	
**Payer**				0.3752
Commercial	2799 (46.4)	4005 (45.7)	6804 (46.0)	
Medicare supplemental	3230 (53.6)	4764 (54.3)	7994 (54.0)	
**Payer product name**				<0.0001
CDHP	275 (4.6)	472 (5.4)	747 (5.1)	
Comprehensive	1219 (20.2)	1929 (22.0)	3148 (21.3)	
EPO	27 (0.5)	38 (0.4)	65 (0.4)	
HDHP	151 (2.5)	241 (2.8)	392 (2.7)	
HMO	790 (13.1)	863 (9.8)	1653 (11.2)	
POS	312 (5.2)	447 (5.1)	759 (5.1)	
POS with capitation	123 (2.0)	135 (1.5)	258 (1.7)	
PPO	3034 (50.3)	4557 (52.0)	7591 (51.3)	

Data are presented as n (%), mean±sd or median (interquartile range). IQR: interquartile range; CDHP: consumer-directed health plan; EPO: exclusive provider organisation; HDHP: high-deductible health plan; HMO: health maintenance organisation; POS: point of service; PPO: preferred provider organisation.

Almost half of the patient population had ≥2 exacerbations at baseline (42.7%), with a mean±sd number of baseline pulmonary exacerbations of 3.0±3.8. The most common respiratory comorbidities (>10%) were COPD (28.5%), dyspnoea (26.2%), cough (25.4%), other lung or airway disease (23.8%), asthma (20.1%), chronic bronchitis (11.2%) and acute bronchitis (10.2%) (table 2). The most common (>10%) non-respiratory comorbidities were hypertension (44.5%), GERD (15.5%), malignancy (11.1%) and ischaemic heart disease (10.5%). Additionally, p-values for comparisons between exacerbation groups (<2 *versus* ≥2) were calculated to help guide the decision of covariates in the multivariable model (table 2).

**TABLE 2:** Comorbidities in the baseline period stratified by exacerbations over the 2-year follow-up period

	**<2 exacerbations**	**≥2 exacerbations**	**Overall**	**p-value^#^**
**Patients n**	6029	8769	14 798	
**Respiratory comorbidities**				
Acute bronchitis	389 (6.5)	1122 (12.8)	1511 (10.2)	<0.0001
Asthma	898 (14.9)	2069 (23.6)	2967 (20.1)	<0.0001
Chronic bronchitis	432 (7.2)	1223 (14.0)	1655 (11.2)​	<0.0001
COPD	1375 (22.8)	2842 (32.4)	4217 (28.5)	<0.0001
COPD and asthma	299 (5.0)	848 (9.7)	1147 (7.8)	<0.0001
Cough	1230 (20.4)	2535 (28.9)	3765 (25.4)	<0.0001
Dyspnoea	1275 (21.2)	2603 (29.7)	3878 (26.2)	<0.0001
Emphysema	236 (3.9)	450 (5.1)	686 (4.6)	0.0006
Haemoptysis	112 (1.9)	228 (2.6)	340 (2.3)	0.0037
Idiopathic interstitial lung disease	330 (5.5)	621 (7.1)	951 (6.4)	0.0001
Idiopathic pulmonary fibrosis	63 (1.0)	107 (1.2)	170 (1.2)	0.3657
Lung transplant^¶^	6 (0.1)	48 (0.6)	54 (0.4)	<0.0001
Malignant neoplasm of bronchus and lung	117 (2.0)	195 (2.2)	312 (2.1)	0.2628
Nasal polyps	18 (0.3)	58 (0.7)	76 (0.5)	0.0035
NTM lung disease	192 (3.2)	471 (5.4)	663 (4.5)	<0.0001
Other lung or airway disease	1121 (18.6)	2407 (27.5)	3528 (23.8)	<0.0001
Pulmonary hypertension	39 (0.7)	88 (1.0)	127 (0.9)	0.0264
Pulmonary fibrosis	330 (5.5)	624 (7.1)	954 (6.5)	<0.0001
Pulmonary tuberculosis	15 (0.3)	16 (0.2)	31 (0.2)	0.4938
**Non-respiratory comorbidities**				
Atrial fibrillation	539 (9.0)	875 (10.0)	1414 (9.6)	0.0373
Cerebrovascular disease	252 (4.2)	442 (5.0)	694 (4.7)	0.0167
Chronic liver disease	95 (1.6)	177 (2.0)	272 (1.8)	0.0564
Chronic kidney disease	432 (7.2)	721 (8.2)	1153 (7.8)	0.0201
Depression	376 (6.2)	798 (9.1)	1174 (7.9)	<0.0001
GERD	728 (12.1)	1568 (17.9)	2296 (15.5)	<0.0001
Heart failure	374 (6.2)	733 (8.4)	1107 (7.5)	<0.0001
Hypertension	2505 (41.6)	4081 (46.5)	6586 (44.5)	<0.0001
Irritable bowel disease	63 (1.0)	119 (1.4)	182 (1.2)	0.1059
Ischaemic heart disease	572 (9.5)	980 (11.2)	1552 (10.5)	0.0011
Malignancy	584 (9.7)	1065 (12.2)	1649 (11.1)	<0.0001
Osteoporosis	340 (5.6)	560 (6.4)	900 (6.1)	0.0669
Peptic ulcer disease	31 (0.5)	50 (0.6)	81 (0.6)	0.7336
Psoriatic arthritis	14 (0.2)	26 (0.3)	40 (0.3)	0.5626
Rheumatoid arthritis	244 (4.1)	435 (5.0)	679 (4.6)	0.0102
Type 2 diabetes	824 (13.7)	1392 (15.9)	2216 (15.0)	0.0002

Data are presented as n (%). COPD: chronic obstructive pulmonary disease; NTM: nontuberculous mycobacterial disease; GERD: gastro-oesophageal reflux disease. ^#^: The p-value is for comparison of the <2 and ≥2 exacerbations groups; ^¶^: p-value was calculated using the Fisher exact test.

During the 1-year baseline period, 28.3% of the overall population had ≥1 all-cause hospitalisation and 30.9% had ≥1 all-cause ED visit. Number of hospitalisations, ED visits and use of interventions such as antibiotics and LABAs at baseline were significantly higher in patients with ≥2 exacerbations compared with those with <2 exacerbations (p<0.0001; table 3). Nearly half of the patients in the ≥2 exacerbations group had a claim for a macrolide or LABA prescription, and 7.6% had multiple macrolide claims for ≥3 consecutive months. Patients with ≥2 exacerbations a year were significantly more likely to receive repeat antibiotic treatment (2 consecutive months; 18.3%) compared with patients with <2 exacerbations (5.2%, p<0.0001) (table 3).

**TABLE 3:** Healthcare utilisation and intervention use in the baseline period stratified by exacerbations over the 2-year follow-up period

	**<2 exacerbations**	**≥2 exacerbations**	**Overall**	**p-value**
**Patients n**	6029	8769	14 798	
**Exacerbations**				<0.0001
** **Mean±sd	1.1±1.8	3.0±3.8	2.2±3.3	
** **Median (IQR)	0 (0–2)	2 (0–4)	1 (0–3)	
**All-cause hospitalisations**				<0.0001
** **Mean±sd	0.44±1.4	0.68±1.8	0.58±1.6	
** **Median (IQR)	0 (0–0)	0 (0–1)	0 (0–1)	
**All-cause ED visits**				<0.0001
** **Mean±sd	0.42±1.1	0.60±1.3	0.53±1.2	
** **Median (IQR)	0 (0–1)	0 (0–1)	0 (0–1)	
**Use of interventions**				
Antibiotics	1956 (32.4)	5136 (58.6)	7092 (47.9)	<0.0001
Repeat antibiotics^#^	312 (5.2)	1608 (18.3)	1920 (13.0)	<0.0001
Chronic antibiotics^¶^	19 (0.3)	141 (1.6)	160 (1.1)	<0.0001
Macrolides	1511 (25.1)	4081 (46.5)	5592 (37.8)	<0.0001
Chronic macrolides^¶^	88 (1.5)	667 (7.6)	755 (5.1)	<0.0001
LABAs	1626 (27.0)	3952 (45.1)	5578 (37.7)	<0.0001
Inhaled steroids	380 (6.3)	757 (8.6)	1137 (7.7)	<0.0001

Data are presented as n (%), mean±SD or median (interquartile range). IQR: interquartile range; ED: emergency department; LABA: long-acting β2 agonist. ^#^: repeat is defined as the same antibiotic for 2 consecutive months; ^¶^: chronic was defined as having at least 90 total treated days in the time period.

### Longitudinal trends in exacerbations and hospitalisations

**FIGURE 2:**
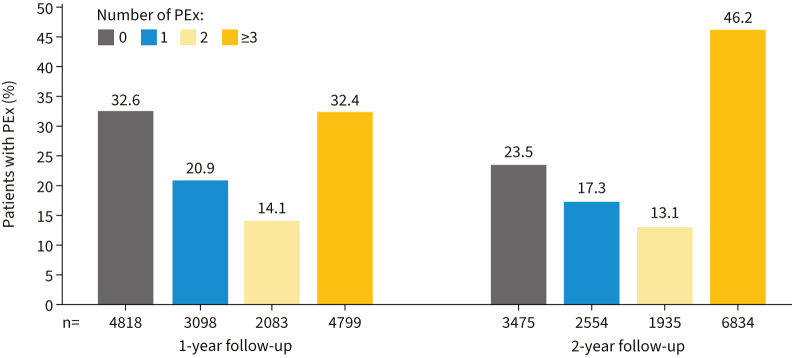
Total occurrences of PEx experienced by patients with bronchiectasis over the 1- and 2-year follow-up periods. Total n=14 798. PEx: pulmonary exacerbations.

The mean±sd number of pulmonary exacerbations in the overall population was 2.5±3.5 in year 1 and 3.9±5.4 in year 2. The overall proportion of patients experiencing ≥1 exacerbation cumulatively increased from 67.4% in the first year of follow-up to 76.6% over the entire 2 years. Similarly, the overall proportion of patients experiencing ≥3 exacerbations cumulatively increased from 32.4% in the first year of follow-up to 46.2% over the entire 2 years (p<0.0001; figure 2). The overall proportion of patients who experienced no exacerbations during the baseline period through year 2 of follow-up was 14.7% (n=2178).

**FIGURE 3:**
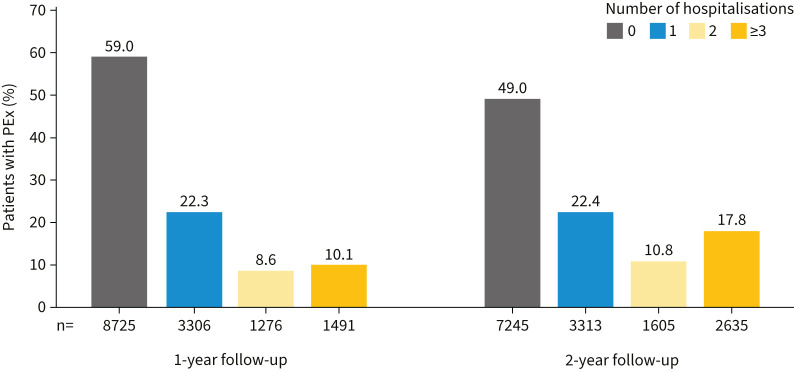
Patients with bronchiectasis who were hospitalised over the 1- and 2-year follow-up periods. Total n=14 798. PEx: pulmonary exacerbations.

At baseline, 28.3% of the overall bronchiectasis population had ≥1 all-cause hospitalisation. The overall proportion of patients experiencing ≥1 hospitalisation cumulatively increased from 41.0% in the first year of follow-up to 51.1% over the 2-year follow-up period. In contrast, the overall proportion of patients experiencing ≥3 all-cause hospitalisations cumulatively increased from 10.1% in the first year of follow-up to 17.8% over the 2-year follow-up period (p<0.0001; figure 3).

### Factors associated with ≥2 exacerbations during follow-up

Unadjusted ORs were calculated to understand the association between number of exacerbations at baseline and in the subsequent 2 years of follow-up. Patients with ≥2 pulmonary exacerbations in the baseline period had 3.4 times greater odds (95% CI 3.1–3.6) of experiencing ≥2 exacerbations during the first year of follow-up, and 3.0 times greater odds (95% CI 2.8–3.2) of ≥2 exacerbations during the second year (table 4). Furthermore, patients with ≥2 pulmonary exacerbations in the first year of follow-up had 3.6 times greater odds (95% CI 3.4–3.9) of experiencing ≥2 exacerbations during the second year of follow-up (table 4).

**TABLE 4:** Cross-tabulation to assess the relationship between exacerbations at baseline or year 1 and exacerbations in year 2

**A. OR of patients with ≥2 pulmonary exacerbations in the baseline period experiencing ≥2 exacerbations during the first year of follow-up**				
**≥2 exacerbations** **(baseline)**	**≥2 exacerbations during first year of follow-up**		**Total**	**OR (95% CI)**
	**Yes**	**No**		
**Yes**	3998 (58.1)	2319 (29.3)	6317	3.35 (3.1–3.6)
**No**	2884 (41.9)	5597 (70.7)	8481	
**Total**	6882	7916	14 798	

**B. OR of patients with ≥2 pulmonary exacerbations in the baseline period experiencing ≥2 exacerbations during the second year of follow-up**				
**≥2 exacerbations** **(baseline)**	**≥2 exacerbations during second year of follow-up**		**Total**	**OR (95% CI)**
	**Yes**	**No**		
**Yes**	2675 (61.3)	3642 (34.9)	6317	2.96 (2.8–3.2)
**No**	1686 (38.7)	6795 (65.1)	8481	
**Total**	4361	10 437	14 798	

**C. OR of patients with ≥2 pulmonary exacerbations in the first year of follow-up experiencing ≥2 exacerbations during the second year of ​follow-up**				
**≥2 exacerbations** ** during first year** ** of follow-up**	**≥2 exacerbations during second year of follow-up**		**Total**	**OR (95% CI)**
	**Yes**	**No**		
**Yes**	2983 (68.4)	3899 (37.4)	6882	3.63 (3.4–3.9)
**No**	1378 (31.6)	6538 (62.6)	7916	
**Total**	4361	10 437	14 798	

Data are presented as n, n (%) or unadjusted OR (95% CI). OR: odds ratio; CI: confidence interval.

The multivariable logistic regression model assessed baseline factors that were associated with having ≥2 exacerbations in the 2 years of follow-up. Covariates, listed in table 5, were selected based on known impacts on bronchiectasis prognosis or number of exacerbations and statistical differences in baseline data between the two number-of-exacerbations groups. The multivariable model had a c-statistic value of 0.71, and a Pr(>Chi) value of <0.0001, demonstrating a strong goodness of fit. The following baseline variables were positively associated with having a greater probability of ≥2 exacerbations in the follow-up period: number of baseline exacerbations, number of ED visits (association not significant), chronic macrolide use, LABA use, or having GERD, heart failure or *P. aeruginosa* (table 5). When adjusted, the odds of ≥2 exacerbations during the follow-up increased by 3% for each additional ED visit at baseline, 31% for each additional exacerbation at baseline and 2.6 times for each patient receiving chronic macrolides at baseline (table 5). Minimum and maximum VIF values were 1.01 and 1.21, respectively, indicating very low correlation among selected independent variables (table 5).

**TABLE 5:** Multivariable logistic regression analysis to assess the relationship between baseline factors and having ≥2 exacerbations in year 2

**Baseline covariate**	**Estimate**	**Standard error**	**OR (95% CI)**	**z-value**	**Pr(>|z|)**	**VIF**
**Age**	0	0	1 (1–1)	–1.75	0.0809	1.04
**Male sex**	–0.1	0.04	0.91 (0.85–0.98)	–2.54	0.0111	1.02
**Number of ED visits**	0.03	0.02	1.03 (1.00–1.07)	1.82	0.0694	1.08
**Number of exacerbations^#^**	0.27	0.01	1.31 (1.28–1.33)	27.01	<0.0001	1.13
**Chronic macrolide use** ^¶^	0.95	0.13	2.6 (2.03–3.33)	7.55	<0.0001	1.11
**LABA use**	0.5	0.04	1.65 (1.53–1.78)	12.87	<0.0001	1.06
**Inhaled steroid use**	0.06	0.07	1.06 (0.92–1.21)	0.8	0.4255	1.02
**Cough**	–0.04	0.04	0.96 (0.88–1.05)	–0.85	0.3965	1.11
**Dyspnoea**	0	0.05	1 (0.91–1.09)	–0.02	0.9878	1.21
**GERD**	0.21	0.05	1.23 (1.11–1.37)	3.99	<0.0001	1.04
**Heart failure**	0.19	0.07	1.21 (1.04–1.40)	2.51	0.012	1.13
**Haemoptysis**	–0.02	0.13	0.98 (0.76–1.26)	–0.15	0.8842	1.01
***Pseudomonas aeruginosa***	0.57	0.2	1.78 (1.21–2.61)	2.93	0.0034	1.01

The model had a goodness of fit c-statistic value of 0.71, and a pr(>Chi) value of <0.0001. OR: odds ratio; CI: confidence interval; VIF: variance inflation factor; ED: emergency department; LABA: long-acting β2 agonist; GERD: gastro-oesophageal reflux disease. **^#^**: during the 1-year baseline period; ^¶^: chronic was defined as having at least 90 total treated days in the time period.

## Discussion

The results from this study demonstrate that in patients with bronchiectasis, frequent exacerbations (≥2) at baseline were associated with more exacerbations, ED visits, all-cause hospitalisations, and both LABA and macrolide use after 2 years of follow-up. Additionally, higher rates of pulmonary exacerbations at baseline were associated with greater occurrence in the future (1–2 years of follow-up). The overall proportion of patients experiencing ≥1 pulmonary exacerbation or hospitalisation increased from year 1 to year 2, with a minority of the overall population experiencing no exacerbations during the study period. These findings add to the evidence currently in the literature, due to the additional use of the IBM® MarketScan® commercial claims database, and an algorithm with unique definitions of bronchiectasis and pulmonary exacerbations.

Our findings are consistent with previous studies in both bronchiectasis and COPD that have similarly shown that patients with a greater number of exacerbations at baseline have a greater risk of future exacerbations and hospitalisations [11, 12, 14]. It is worth noting that the algorithm used in this study to identify exacerbations is consistent with studies on economic burden of exacerbations in COPD and cystic fibrosis [13, 15]. For example, Chalmers *et al.* (2018) [12] demonstrated that frequent exacerbations were the strongest predictor of future exacerbations in patients with bronchiectasis; incidence rate ratios for future exacerbations increased from 1.73 for patients with one baseline annual exacerbation, to 3.14 for patients with two baseline annual exacerbations. In a systematic literature review of resource use and costs associated with bronchiectasis, mean annual baseline hospitalisation rates of 0.3–1.3 were reported, which is consistent with the mean 0.58 baseline annual hospitalisations in the present study [11]. Furthermore, significant increases in ED visits and physician office visits in patients with frequent exacerbations were found [11]. A higher Bronchiectasis Severity Index score is also indicative of a greater number of exacerbations and is associated with a greater risk of mortality [8].

Factors that are associated with patients with bronchiectasis having ≥2 pulmonary exacerbations include having a greater number of ED visits, having a greater number of exacerbations at baseline, chronic macrolide use, LABA use, having GERD and the presence of *P. aeruginosa*. Furthermore, most of the target population experienced ≥2 exacerbations over the 3 years of study, with very few experiencing none. Therefore, it is likely that at least one of these characteristics will impact patients with bronchiectasis. Future studies may consider these key patient characteristics when deciding on clinical trial inclusion criteria, since patients with frequent exacerbations may benefit the most from treatment aimed to reduce exacerbations.

As macrolides are the recommended treatment for patients with frequent exacerbations, this may account for the finding that higher macrolide use at baseline is associated with increased number of exacerbations [3]. These results are not necessarily indicative of a causal relationship, since macrolides are used to treat exacerbations and all claims for macrolides were used in the analyses and were not separated by the indication.

*P. aeruginosa* commonly colonise the airways of individuals with bronchiectasis [16]. The finding that the presence of *P. aeruginosa* at baseline is related to increased exacerbations is consistent with data collected from a prospective study by Chawla *et al*. (2015) [16] which showed that patients with both bronchiectasis and *P. aeruginosa* colonies had a higher number of exacerbations. Additional research is required to identify the mechanisms of *P. aeruginosa* that may account for this association. In contrast, NTM was not associated with increased exacerbations; this may be because patients with NTM at baseline are already receiving active therapies to treat infections, which overlap with the treatment of both acute and frequent exacerbations. However, it is important to note that this study neither directly investigated whether patients with NTM were being treated nor accounted for potential patients with NTM who were not listed in the claims databases.

Similarly, causal relationships cannot be inferred between incidence of exacerbations, and the other variables of the multivariate model: having a greater number of both ED visits and exacerbations at baseline, having GERD and LABA use. Incidentally, however, previous research has also associated such variables with having a higher number of exacerbations [11, 12, 17–21]. Of particular interest, comorbidities with asthma and COPD, which are proxied by LABA use, have the most established relationship with increased exacerbations in the literature [18, 19, 21]. However, it should be noted that in contrast to our findings, an earlier prospective observational study in a Spanish cohort by Menéndez *et al.* (2017) [20] showed that the number of previous exacerbations was not significantly associated with future hospitalisations. This emphasises the importance of further research into the associations of these variables with having ≥2 pulmonary exacerbations to better understand the factors that lead to frequent exacerbations, which could improve our understanding of ways to prevent them.

A potential limitation of this study is that Medicare data may not be generalisable for all patients ≥65 years of age because the majority of data are only captured for those who are still employed. Additionally, inherent to claims data research, the clinical accuracy of the coding could not be assessed, and symptoms related to exacerbations are not captured. In addition, patients with health maintenance organisation or full or partial capitated point-of-service insurance coverage were excluded from this study because financial information for this population was incomplete. Furthermore, small- and medium-sized insurance firms may be underrepresented in the sample, as most data come from larger employers. No information was available for disease severity and intervention use outside of hospitals. Data not captured in claims, such as smoking status, disease severity and the presence of respiratory comorbidities, are a potential source of confounding known to claims data. Moreover, findings were based on retrospective claims data, which have inherent limitations. Lastly, causal relationships could not be derived from the associations found using the multivariate model developed in this study.

Strengths of these data and method employed include that COPD events and pulmonary exacerbations were separated, ensuring that only pulmonary exacerbations were included. Additionally, unlike previous studies using claims databases [5, 11, 12], the present study did not use ICD-9/10 codes alone to identify individuals with bronchiectasis and pulmonary exacerbations. Instead, an algorithm with unique definitions of bronchiectasis and pulmonary exacerbations was developed that may better reflect the true frequency of exacerbations [11]. Furthermore, previous studies have only used the Medicare database to capture outpatient claims, whereas this study also uses the IBM® MarketScan® claims database to capture additional data from a commercially insured population that otherwise have not been characterised [13, 22–24]. This study was also consistent with how studies on the economic burden of exacerbations have been operationalised in COPD and cystic fibrosis [15, 25]. Moreover, the latest data available in the USA were utilised to perform a detailed characterisation of people with bronchiectasis. Lastly, potential confounding variables were accounted for by using proxies to categorise respiratory comorbidities, such as utilising prescription paid claims for LABA to categorise COPD and asthma rather than relying on ICD-9/10 codes alone.

### Conclusions

Overall, our findings improve our understanding of characteristics that may increase the number of exacerbations in patients with bronchiectasis, provide insights that could support the design of future clinical trials and highlight the need to address the prevention of exacerbations as part of bronchiectasis management. The longitudinal trends of increasing pulmonary exacerbations and hospitalisation rates observed over the 2-year follow-up period suggest that frequent exacerbations in patients with bronchiectasis may increase the likelihood of future exacerbations. In future work it would be interesting to assess the overlap between factors associated with ≥2 exacerbations at year 1 and year 2, to determine if additional factors may influence progression of the disease.
